# The complete chloroplast genome of *Zanthoxylum acanthopodium* DC. (Rutaceae) and its phylogenetic analysis

**DOI:** 10.1080/23802359.2020.1823268

**Published:** 2020-10-27

**Authors:** Hai-Ling Li, Nong Zhou, Dong-Qin Guo

**Affiliations:** a College of Biology and Food Engineering, Chongqing Three Gorges University, Chongqing, China; b Engineering Laboratory of Chongqing for Green Planting and Deep Processing of Genuine Medicinal Materials in Three Gorges Reservoir Area, Chongqing Three Gorges University, Chongqing, China

**Keywords:** complete chloroplast genome, *Zanthoxylum acanthopodium*, characteristics, phylogenetic analysis

## Abstract

We sequenced the complete chloroplast genome of *Zanthoxylum acanthopodium* via high-throughput sequencing technology, and analyzed its structural characteristics and phylogenetic relationships. The chloroplast exhibits a genome length of 158,473 bp, including a pair of inverted repeat regions (IRa and IRb) of 27,369 bp, a small single-copy (SSC) region of 17,629 bp and a large single-copy (LSC) region of 85,570 bp. The annotation analysis identified 112 genes, containing 78 protein-coding genes, 30 tRNA genes and 4 rRNA genes. The phylogenetic analysis showed that *Z. acanthopodium* was closely related to *Z. piperitum* and *Z. tragodes*.

The *Zanthoxylum* genus belongs to the Rutaceae family. Plants in this genus are mainly utilized as spices and medicinal agents. About 250 species distributed in tropical and temperate regions of Asia and North America. In southern Yunnan and southeast Tibet of China, *Zanthoxylum acanthopodium* is a small tree with a height of 4.0 m, and grows in mountain shrubs or sparse forests at an altitude of 1,400–2,500 m (Editorial Committee of Chinese Journal of Plant of Chinese Academy of Sciences 1997). Its fruit is widely used as a spice or additional flavor for traditional delicacy. *Z. acanthopodium* was extensively used in traditional folk medicine and possess anti-inflammatory, antibacterial properties (Yanti et al. 2011; Majumder et al. 2014; Bhatt et al. 2018). However, there have been no genomic studies on *Z. acanthopodium.* Herein, we reported and characterized complete chloroplast genome of *Z. acanthopodium*, which will provide more sequences information to investigate the genome and phylogenetic relationship of the Rutaceae family.

The experimental materials of *Z. acanthopodium* were collected from Dali county, Yunnan province, China (100°15′42.76″E, 25°31′33.38″N). The voucher herbarium specimen (No. ZSY111) were deposited at the Herbarium of Medicinal Plants and Crude Drugs of the College of Pharmacy and Chemistry, Dali University. High-quality genomic DNA was extracted using modified CTAB method (Doyle 1987; Yang et al. 2014). Whole-genome sequencing was conducted with pair-end (2 × 300 bp) library on Illumina Hiseq 2500 (Novogene, Tianjing, China) platform. To decrease the redundant data, the original reads were filtered by Trimmomatic v.0.32 software with default parameters (Bolger et al. 2014), the obtained clean reads were assembled into circular contigs using GetOrganelle.py (Jin et al. 2018) with *Z. simulans* (No. NC_037482) as the reference. Finally, the complete chloroplast genome sequences of *Z. acanthopodium* were annotated using Geneious R11.0.2 (Kearse et al. 2012), and then submitted to GenBank with the accession number of MT795653. To investigate its phylogenetic position within family Rutaceae, the complete chloroplast genome sequences of *Z. acanthopodium* and 20 related species from NCBI with *Ailanthus altissima* as an outgroup were aligned using MAFFT V.7.149 (Katoh and Standley 2013). Then, the maximum likelihood tree was generated by RAxML (Stamatakis 2014) with 1000 bootstrap replicates under the GTRGAMMAI substitution model.

The chloroplast genome of *Z. acanthopodium* was 1,58,473 bp in length with a typical quadripartite structure, containing a large single copy-region (LSC, 85,570 bp) and a small single copy-region (SSC, 17,625 bp), which were separated by two inverted repeat (IRa/IRb, 27,639 bp). The genome contained 112 genes, including 78 protein-coding genes, 30 tRNA genes, and 4 rRNA genes. The overall GC content of the genome is 38.5% and those in LSC, SSC, and IR regions were 36.9%, 33.5%, and 42.5%, respectively. A maximum likelihood phylogenomic analysis showed that *Z. acanthopodium* was evolutionarily closest to *Z. piperitum* and *Z. tragodes* (Figure 1).

**Figure 1.**
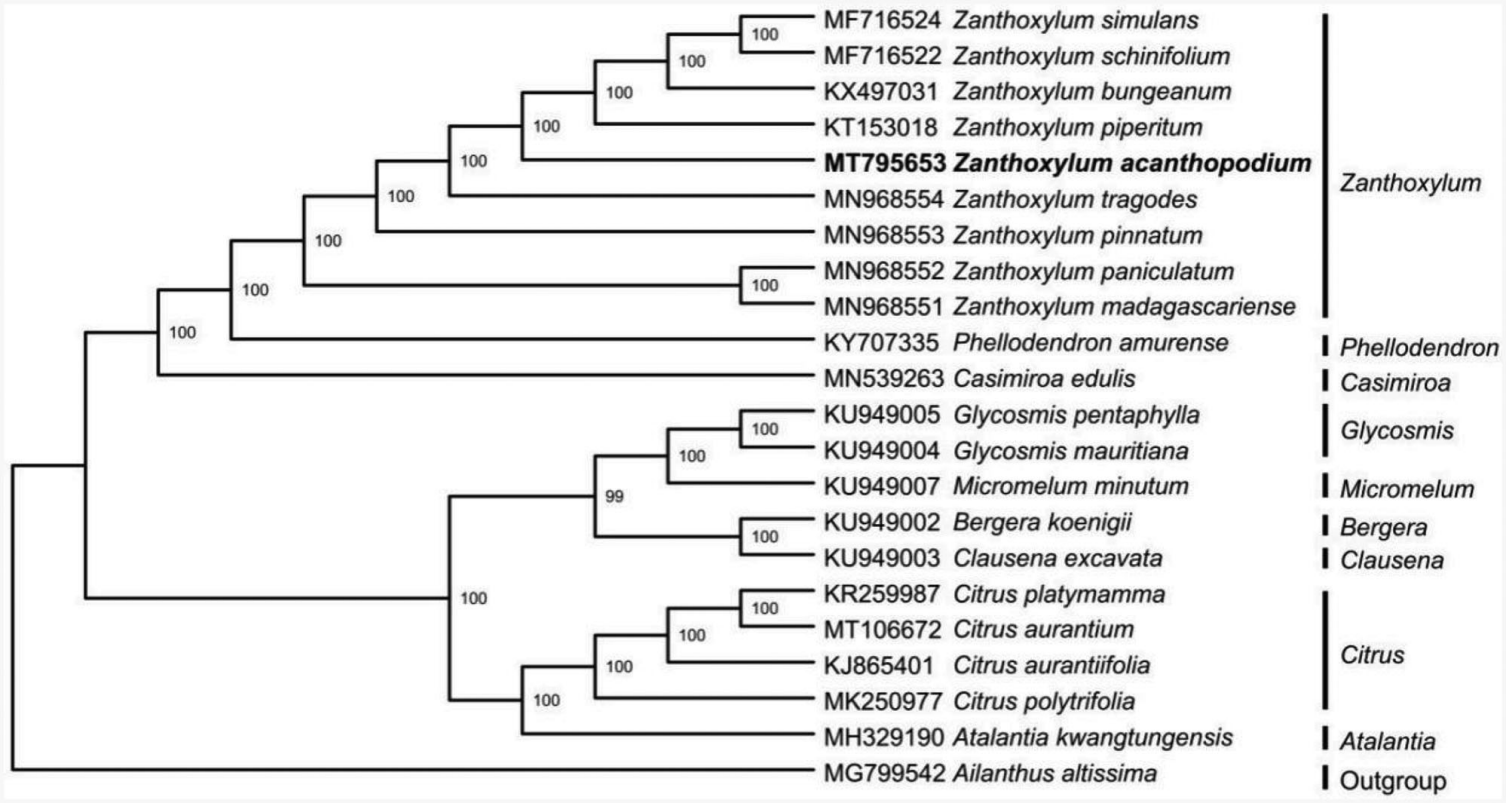
ML phylogenetic tree showing the phylogenetic position of *Zanthoxylum acanthopodium* based on the complete chloroplast genome sequences of 22 species. Bootstrap support values (1000 replicates) are shown next to the nodes.

## Data Availability

The data supporting the finding of this study is available in GenBank. The accession number is MT795653: https://www.ncbi.nlm.nih.gov/nuccore/MT795653.
